# Generation Y New Zealand Registered Nurses’ views about nursing work: a survey of motivation and maintenance factors

**DOI:** 10.1002/nop2.16

**Published:** 2015-04-07

**Authors:** Isabel Jamieson, Ray Kirk, Sarah Wright, Cathy Andrew

**Affiliations:** ^1^ Department of Nursing and Human ServicesChristchurch Polytechnic Institution of TechnologyTe Mātāpuna o Te MātaurakaPO Box 540Christchurch8140New Zealand; ^2^ School of Health SciencesUniversity of CanterburyTe Whare Wānanga o WaitahaChristchurch8140New Zealand; ^3^ Department of ManagementUniversity of CanterburyTe Whare Wānanga o WaitahaChristchurch8140New Zealand

**Keywords:** Career, generation Y, Herzberg, motivation, nursing, work

## Abstract

**Aim:**

The aim of this article was to report on the analysis of qualitative, open text data, received from a national on‐line survey of what factors Generation Y New Zealand Registered Nurses wish to change about nursing and consideration of the potential policy and practice impacts of these requests on their retention.

**Background:**

Prior to the economic recession of 2007–2010, the growing shortage of nurses in New Zealand presented a serious concern for the healthcare workforce. Given the ageing New Zealand nursing workforce, an ageing population and the increasing demands for health care, it is imperative that issues of retention of Generation Y nurses are resolved prior to the imminent retirement of more experienced nurses.

**Design:**

A descriptive exploratory approach using a national wide, on‐line survey, eliciting both quantitative and qualitative data was used.

**Method:**

The survey, conducted from August 2009–January 2010, collected data from Generation Y New Zealand Registered Nurses (*n *=* *358) about their views about nursing, work and career. Herzberg's Motivation‐Hygiene theory was used as the framework for the analysis of the open text data.

**Results:**

The factors that nurses wanted changed were skewed towards Herzberg's hygiene‐maintenance factors rather than motivating factors. This is of concern because hygiene‐maintenance factors are considered to be dissatisfiers that are likely to push workers to another employment option.

## Introduction

In New Zealand, as in other nations around the globe, the growing shortage of nurses in the healthcare workforce presents a problem of increasing significance. At a time when the New Zealand population is ageing and the demands for healthcare services are increasing, the healthcare professional workforce, and in particular nursing, faces a shortage of skilled professionals (Zurn *et al*. 2005, Young & Twinn, 2006, Massey University 2007, New Zealand Nurses Organisation 2007a,b). This being the case, the retention of Generation Y nurses in the healthcare workforce is of vital importance especially as more experience nurses begin to retire.

Further, it has been suggested that members of the Generation Y cohort will have several careers in their lifetime (Leeming 2007). Moreover, Leeming noted that many of these career options have yet to be invented. If this prediction were to prove correct, then it is important to know the future career plans of newly graduated registered nurses so that plans might be put in place, by employers of these nurses, to contribute to their retention in the healthcare workforce and to include them in the future development of the nursing profession. However, there is a lack of literature about Generation Y New Zealand nurses about who they are, why they choose nursing as a career and whether they plan to remain in the nursing profession (Clendon & Walker 2011).

### Background

Given the current shortage of nurses (Kimball & O'Neil 2002, Cassie 2008) and predicted future shortages (Buerhaus 2009, AMN Healthcare 2011) it is imperative that the nursing profession retains its new graduates who are predominantly from the Generation Y cohort. It is costly for the individual to qualify as a registered nurse, both financially and personally. In turn, it is costly for the government, taxpayers and education providers to offer undergraduate nursing courses, and recruitment and retention of employees is a costly endeavour for employers. To ensure the best use of resources, it is in the interests of the key stakeholders, the students, their teachers and their employers, to understand why young people in the 21st century are still choosing to qualify as nurses given that: More career choices for women are available; historically, nursing has been a poorly paid profession and there are predictions that Generation Y workers will opt to have several careers in their life time.

Therefore, it is imperative to understand what has influenced the career choice of Generation Y nurses. Are they just ‘passing through’ on their way to bigger and better careers? Or is nursing still seen as a good career choice, especially for women, and is this nursing's saving grace?

Given the lack of available literature and empirical data about Generation Y New Zealand nurses and their views about nursing, the research question was: What are the views of Generation Y New Zealand Registered Nurses towards nursing, work and career? The aims of this research were to determine: (1) what motivated this generation to choose nursing as a career option? (2) what are the future work and career plans of these nurses? (3) how long do these nurses intend to stay in the nursing profession? (4) what intrinsic and extrinsic factors influence these nurses to either remain in or exit from the healthcare workforce? The focus of this article is to report on the concerns expressed by the Generation Y nurses about the professional of nursing and how ignoring these concerns may contribute to an exodus of this cohort of nurses at the very time when their retention is critical.

## Literature review

### The meaning of work

The definition of the term ‘work’ is complex. Defined as a noun, work may be considered an activity whereby one is expected to apply either sustained physical or mental force so that a task may be performed (Work 2008a). Additionally, the term ‘work’ may be used to describe one's place of employment and/or the duties that one undertakes there (Work, 2008b). Work is also defined as a ‘task to be undertaken…thing done or made by work; result of an action’ (Deverson, 1997, p. 1231). Thus, work has several meanings.

Gardner *et al*. (2001) expressed the view that ‘good work’ can be defined as occurring when individuals are concerned about the implications and the impact of their work for the wider world and vice versa. Gardner *et al*. (2001) suggested that individual workers and employers have the power to define what good work is. Miller (2006) suggests that good work in nursing can be considered to be work that is both ‘technically and scientifically effective and morally and socially responsible’ (p. 471) and is influenced by factors such as: mentors, the workplace environment and personal values.

Work also affords us status, or lack of status, in the society where we live. This is evident by the clear link between the type of work, society's view of that work and the monetary rewards afforded, for example, the status of a driver vs. a lawyer (Doyle 2003, Landy & Conte 2007). Regardless of occupation, Landy and Conte (2007) noted that the majority of adults in the Western world dedicate more of their time to work than to any other activity. Given this allocation of time to work, it can be considered to be significant, fundamental and important aspect of adult life (Greenhaus *et al*. 2000, Landy & Conte 2007).

## Work as a determinant of health

Compelling evidence has suggested that being able to work is good for both personal and community health (Wilkinson & Marmot 2003). Work contributes to a sense of status, self‐worth and well‐being and increases life expectancy. Participants of a New Zealand project exploring fairness at work noted that ‘work is a critical source of well‐being and identity expressed by the whakataukī: Mauri Mahi, Mauri Ora, Mauri Noho, Mauri Mate: a working soul is a healthy soul’ (Human Rights Commission 2010, p. 3). Furthermore, people flourish when they have autonomy over their work. However, work and the workplace can be ‘a doubled edge sword’. The demands of the job and the workplace can put workers under considerable strain, with excessive stress and unsafe conditions being a precursor to poor health and a shortened life expectancy. Other contributing factors to the poor health of workers are having little opportunity to use skills, lack of autonomy and unequal rewards for effort expended. Moreover, the psychosocial culture of the workplace also plays an important part in the health of workers (Wilkinson & Marmot 2003, Black 2008, The Australasian Faculty of Occupational & Environmental Medicine 2010, Wilkinson & Pickett 2010).

### Work‐life balance

The concept of having a work‐life balance is viewed by many workers as an important issue for them (Hays Specialist Recruitment Limited 2010, Jamieson *et al*. 2013). It has been suggested that work‐life balance is what Generation Y value most in a work environment (McCrindle 2006, McCrindle & Pleffer 2008). They will not view work as their ‘life’, rather they will favour flexibility in the workplace (Jamieson *et al*. 2013). Further, Generation Y nurses note that they want shift patterns that complement their out of work‐life activities with workloads that do not leave them exhausted for their off work time (Jamieson *et al*. 2013).

### Herzberg Motivation‐Hygiene Theory of motivation to work

The Motivation‐Hygiene Theory was developed following in‐depth interviews of 200 American engineers and accountants based in the Pittsburgh area during the late 1950s (Herzberg *et al*. 1959). It was further refined by Herzberg in the 1960s (Herzberg 1968) and has been replicated innumerable times. The aim of Herzberg *et al*. (1959) research was to discover individual workers’ attitudes towards their work and what it was that individual workers wanted from their jobs.

### Push‐pull factors

The Motivation‐Hygiene Theory, proposes that two sets of independent and distinctive factors exist which serve to motivate workers. Motivational factors or ‘motivators/satisfiers’ are intrinsic factors which relate with the content of the job. These ‘pull factors’ are likely to be mentioned by workers as reasons to stay in their job (Navigate 2002). Motivators contribute to personal growth and long‐lasting changes of attitudes and are more likely to contribute to increased job satisfaction. Consequently, motivating factors become a source of job ‘satisfiers’. The motivating factors are: achievement, recognition, work itself, responsibility, advancement and personal growth with the most important motivators being work itself, responsibility and advancement (Herzberg *et al*. 1959, Herzberg 1968, 1993, 2008) .

Hygiene factors, or maintenance factors and dissatisfiers, are extrinsic to the worker and relate with the context of the job. These factors are likely to be ‘push factors’ or reasons for leaving a job (Navigate 2002). Hygiene factors prevent dissatisfaction with the job but do not contribute to long term job satisfaction. Hygiene factors are more likely to contribute to dissatisfaction. Hygiene factors include: status, security, relationship with subordinates, personal life, relationship with peers, salary, work conditions, relationship with supervisor, company policy and administration and supervision. All factors are equally important, but some may become more important than others depending on circumstances (Herzberg *et al*. 1959, Herzberg 1968, 1974, 1993). Hygiene factors are so named because these factors were considered by Herzberg *et al*. (1959) to be the factors that ‘prevent’ dissatisfaction or discontent at work just as medical hygiene measures ‘prevent’ disease transmission. Herzberg *et al*. (1959) suggested that employees expect the hygiene factors to exist in the workplace; therefore, hygiene factors are not in and of themselves motivators for work. Hygiene factors serve as a basis for a satisfied employee and are not themselves satisfiers. Positive changes in hygiene factors will only result in short term attitude changes. Herzberg *et al*. (1959) suggested that hygiene factors fail as satisfiers because they do not contribute to the workers’ personal growth. The fewer motivational factors that exist in a job, the more important it is that hygiene factors are in place to prevent dissatisfaction.

When hygiene factors are considered by workers to be poor or non‐existent, the result is job dissatisfaction. When hygiene factors are considered to be positive, the result is the absence of dissatisfaction rather than existence of satisfaction (Herzberg *et al*. 1959). Job satisfaction occurs when the individuals needs for self‐actualization occur. Herzberg *et al*. (1959) concluded that: (1) work is the most important aspect of who we are; (2) work conditions do not have the potential to provide satisfaction; (3) rewards generated from the performance of the work itself contribute to motivation and job satisfaction while factors external to the work itself, that is hygiene factors, prevent job dissatisfaction. Moody and Pesut (2006) noted that Herzberg *et al*. (1959) theory has:

Been used to explain motivation in health care contexts and nursing work. Significant correlations among nurses’ work motivation, nurses’ internal psychological states and external job characteristics such as autonomy, work conditions, quality of supervision and interpersonal relations have been reported for staff nurses…nurses’ work motivation is significantly and positively related with both the quality of job content and to personal meaning. (p. 25)

### Generation Y

Sociologists define generations as a group of age related individuals who were born during the same period in time. The span of an age related generation is approximately 15–20 years. In the mid twentieth century Karl Mannheim, an Austro‐Hungarian sociologist and historian, noted that people positioned in the same generational cohort may hold different views about the world around them than previous generations. It was Mannheim's observation that the unique experiences of each generational cohort contributed to social change (Mannheim 1952). For example, Elder (1999) suggested that a generation raised in a time of economic hardship will hold a very different view about life than those living in a time of prosperity.

There are several definitions of the age span of Generation Y in the literature, for the purposes of this paper Generation Y are consider to be those individuals born between the years of 1980–1994. The age ranges used here is reflective of McCrindle and Pleffer (2008) work.

This particular cohort, the children of the Baby Boomers, have been the subject of much research, especially in the management and marketing fields. Sheahan (2005) described Generation Y as mature, resilient, fast learners who demonstrate both practical and enterprising skills. He suggests that this is because many of this group grew up with either divorced parents, with whom they lived week about with, or with two working parents who were focused on career development and long work hours.

### Generation Y attitudes to work

From a workforce perspective, McCrindle Research Company (McCrindle 2006, 2007, McCrindle & Pleffer 2008) suggested that there are five key elements that Generation Y will value most in a work environment. Firstly, work‐life balance matters. They will not view work as their ‘life’, rather they will favour flexibility in the workplace. Secondly, they value the culture of their workplace, placing a high importance on being socially connected with peers. Thirdly, this cohort likes change and hence seeks a workplace that offers variety. Fourthly, Generation Y prefers managers who not only communicate well with them but also offer mentorship. Finally, McCrindle (2006, 2007) suggested that Generation Y place a high worth on a workplace that offers them ongoing education.

Others, such as Healy (2008) suggested that Generation Y are looking for a career that they can be passionate about. Healy (2008) also suggests that Generation Y employees are computer savvy, keen to be team players and are results orientated. Importantly, Healy (2008) suggested that Generation Y employees want ongoing mentorship and regular feedback on their performance. Hershatter and Epstein (2010) agree that Generation Y want and need support and mentorship in the early days of their work and will do well in the long term if they are well supported in the short term.

However, little empirical research has been published to date about Generation Y views and values related with work (Families and Work Institute 2005, Deal *et al*., 2010, Kowske *et al*. 2010). Deal *et al*. suggested that ‘the relatively sparse empirical research published on Millennials [Generation Y] is confusing at best and contradictory at worst’ (p. 191) and cautioned that although generational differences do exist the ‘differences are often modest at best’ (p. 196). Kowske *et al*. (2010) examined data collected over an 18‐year period, via the Kenexa WorkTrends USA employee opinion survey (*N* = 115,044). The large dataset for this research contributes significantly to validity of the results. Data were analysed for generational effects on attitude to work. Results suggested that while some different views about work are apparent across generations, the differences were minimal. Generation Y were likely to report ‘higher levels of overall company and job satisfaction, satisfaction with job security, recognition and career development and advancement, but reported similar levels of satisfaction with pay and benefits and the work itself and turnover intentions’ (p. 265). Similarly, research results from New Zealand research of 504 employees across different fields of work noted ‘fewer than expected’ differences between Baby Boomers, Generation X and Generation Y about work values (Cennamo & Gardner 2008, p. 904). However, Cennamo and Gardner noted that Generation Y employees are more likely than others to have a preference for ‘a psychological contract with the organisation which emphasises freedom, status and social involvement’ (p. 904).

An international survey of 3200 Generation Y finance professionals from 122 countries discovered that opportunities for career development and learning opportunities were the key drivers for this group when seeking employment. Career development needs to include the opportunity for a flexible career path and job rotation (Association of Charted Certified Accountants & Mercer 2010). Psychologists Lipkin and Perrymore (2009) have suggested that Generation Y workers may appear overconfident, with an inflated sense of self‐worth, due to overly supportive parenting providing them with constant feedback about their talents. Additionally, they were schooled in a system that promoted concepts of fair play and an ethos of ‘everyone's a winner’ which has resulted in Generation Y workers finding critique or criticism of their abilities by co‐workers or bosses difficult to reconcile. They may lack a sense of ownership of the consequences of poor decision making in the workplace and lack the ability to learn from their mistakes. Lipkin and Perrymore (2009) also noted that Generation Y workers are mostly extrinsically motivated by recognition and rewards and hence will look for tangible recompense such as praise, immediate feedback for a job well done and ongoing acknowledgment of their work. Lack of these rewards may result in insecurity, frustration and decreased performance.

## The study

### Design

This research used a descriptive exploratory approach (Burns & Grove, 2009) using a New Zealand wide on‐line survey to elicit both quantitative and qualitative data.

### The instrument

No one survey was located in the literature that would suitable answer the aims of this research. Accordingly, four instruments (or part thereof) were used for this research to answer the research question and aims.

The instrument, called the 2009/2010 Gen Y nurses survey, comprised eight sections:

1 Demographic data
  - This section comprised 18 mostly closed answer questions.
  - The questions for this section were taken from the NCNZ Annual Practising Certificate survey. This allowed for easy comparison with NCNZ data with regard to ethnicity, length of time working as nurse, hours of work, area of employment and practice.
2 Future career intentions
  - This section comprised 15 questions with a mixture of open and closed questions. One questions related with the views of Generation Y towards career. This question was a 5 point Likert scale asking participants to rate their views from ‘very important’ to ‘not important’ about nine aspects of work such as challenging work and access to education. This section of the survey was used, with permission, from a survey developed by the Australian human resources group AH Revelation (AH Revelations 2006).
3 Career commitment
  - This section comprised seven questions, using a 5 point Likert scale from ‘strongly agree’ to ‘strongly disagree’. These questions related with career commitment were taken from a validated survey designed by Blau (1985, 1988, 1989).
4 Decision to become a nurse
  - Sections four to eight were taken from a survey developed by (McCabe *et al*. 2003) The aim their research was to report on the ‘characteristics, attitudes and employment participation plans of practising RNs in Western Australia’ (2003, p. 2). Permission was received from the authors to adapt their survey.
  - This comprised 22 questions with a 5 point Likert scale from ‘very important’ to ‘not important’.
5 Working environment
  - There were 19 questions in this section with a 5 point Likert scale from ‘strongly agree’ to ‘strongly disagree’.
6 Satisfaction with nursing
  - This section had 15 questions with a 5 point Likert scale from ‘extremely satisfied’ to ‘unsatisfied’.
7 Attitudes to nursing
  - This section had 10 questions with a 5 point Likert scale from strongly agree’ to ‘strongly disagree’.
8 Final comments
  - This section gathered open text data from the open ended question: If there was one thing you could change about nursing what would it be?

The focus of this article is to report on the qualitative free text data from section eight. The open ended question allowed the participants to express their views in their own words.

Demographic data and the results from section one to seven have been reported elsewhere (Jamieson *et al*. 2012, 2013).

### Participants

Emails inviting participation in a survey were sent via the Nursing Council of New Zealand (NCNZ) to 454 eligible participants. The role of the Nursing Council of New Zealand (NCNZ) is to protect the public by setting standards for nursing in New Zealand by accrediting and auditing nursing education programmes and issuing New Zealand Registered Nurses (RNs) with their competency based Annual Practicing Certificates. Therefore, all RNs working in New Zealand must be registered with NCNZ.

Eligible participants were registered nurses born between 1981–1988 (Generation Y) who had supplied an email address to the NCNZ and had indicated that they were willing to participate in surveys via emails. This recruitment method elicited 295 responses = 65% return rate.

To further increase the return rate Nurse Entry to Practice (NETP) coordinators at all New Zealand District Health Boards (DHBs) were contacted by a research assistant and asked if all eligible nurses, both enrolled in a current NETP programme and those who had since completed the programme, could be invited to undertake the survey via an email from the research assistant. All District Health Boards in New Zealand offer a thirteen month NETP programme to support graduate nurses wishing to work in hospitals, primary care or aged/residential care therefor this was considered a useful way of contacting other eligible nurses, as the majority of new registered nurses are enrolled in a NETP for their first year of practice. NETP coordinators from all 21 DHBs agreed to contact eligible RNs. Although two different samples of nurses were recruited, no differences were noted between them because no data were collected about which DHB they worked for; therefore, it was not possible to know which NETP they were enrolled in.

Those interested in undertaking the survey were asked to contact the research assistant via email. The research assistant and a NCNZ staff member checked that there were no duplications. Eighty‐two nurses asked to be sent a link to the survey. Sixty‐three responses were received = 77% return rate. The total numbers of responses equals 358 (*N* = 358). Data collection occurred from August 2009–January 2010. No incentives were offered for participation. Completion and submission of the survey was considered as consent to participate.

Although two different samples of nurses were recruited it is not considered that there were any differences between the cohorts because all nurses in New Zealand must be registered with the NCNZ hence nurses recruited via the NETP are a subset of the larger group. Therefore, the sample of 358 respondents were considered to be one cohort.

### Data collection

Data were collected via the on‐line survey and automatically populated into an Excel spread sheet.

### Ethical considerations

Research Ethics Committee approval was sought and obtained by a university. The on‐line survey opened with an information and consent page which explains that the email recipient was being invited to participant in this research because they were a NZRN born between the years of 1980–1988. They were provided with information about their anonymity, ethics approval and how to contact the researcher and her supervisors if they had any questions. It was noted that completion and submission of the survey was accepted as informed consent for this study. Respondents could chose not to complete the survey once they had commenced.

### Data analysis

NVivo 8 was used as a text management system to code the free text data (QSR International (Americas) Inc 2012). Herzberg's theory was used as a framework for the analysis of the qualitative data, hence free text data were coded against Herzberg's themes of ten hygiene/maintenance factors and six motivators.

Many comments received consisted of one or two words or symbols such as ‘pay’, ‘more money’ or ‘$$$$’ while other replies consisted of entire paragraphs. Given this and because it was not possible to re‐question respondents to further explore their views, a manifest content analysis was conducted using content analysis concepts as described by Graneheim and Lundman (2004). However, it is important to note that ‘a text always involves multiple meanings and there is always some degree of interpretation when approaching a text’ (Graneheim & Lundman 2004, p. 106). As such, it is not possible to be totally objective when coding free text data. The analysis process for the open text data was as follows:

- The final categories for the content analysis were predetermined to be Herzberg's motivation and hygiene/maintenance factors.
- Free text submitted by respondents to the open ended survey question was selected as the unit of analysis.
- The free text was read through by the researcher several times for the researcher to become familiar with the entire text and to obtain a first impression of the emerging meaning units and categories.
- The free text document was prepared for the NVivo programme and uploaded to NVivo 8 for further analysis.
- Meaning units of keywords and phrases that corresponded to Herzberg's framework were highlighted.
- Abstraction was undertaken, by the researcher and overseen by one of supervisors, with a small portion of the text (replies from 50 nurses) to pre‐test that the proposed categories were evident in the text.
- Following this all free text data were abstracted to Herzberg's categories and sub‐categories.
- Subcategories were further divided into groups of related subcategories.
- One of the supervisors checked the researcher's work to ensure abstraction was correct.

### Validity and reliability

Given that no one instrument in its entirety was located in the literature that matched the exact intent of the research aims, it was decided to combine different aspects of the Nursing Council of New Zealand (NCNZ) Annual Practising Certificate (APC) survey, Blau's career commitment scale (1989) and the McCabe *et al*. (2003) 2002 RN survey into one instrument. For details about validity and reliability, see Jamieson *et al*. (2012).

## Findings

### Socio‐demographic characteristics of respondents

The majority of respondents were female, New Zealand European, who had first registered in New Zealand. These results mirror data from the New Zealand Nursing Council.

Their mean age was 25 years, with the majority having worked as registered nurses for <1 year with many having worked for 1–4 years. The majority worked in main urban areas and were employed in public hospitals with the most common clinical areas to work included surgical wards, medical wards, child health areas and perioperative services. The mean hours of work per week were 38.

### Changes that Generation Y New Zealand Registered nurses want for their profession

**Figure 1:**
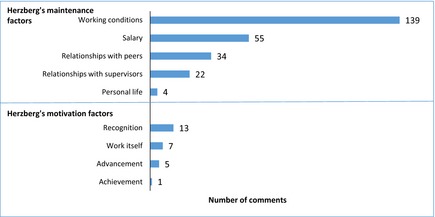
Comments received from the open ended question: what one thing would you change about nursing? *n *=* *271. Responses total >271 as each respondent could report multiple responses.

Respondents were asked the question: what one thing would you change about nursing? Comments were received from 76% of the respondents (*n *=* *271). Overall, more comments were received about Herzberg's maintenance factors (254 comments) than motivation factors (26 comments, Figure 1). Examples of the respondents’ views are detailed in Table 1. There was no restriction on the amount of text that respondents were able to submit.

**Table 1:** Individual respondents’ views about aspects of nursing they would like to change (*n = *271).

Subcategories	Example/meaning unit
Category: maintenance factors	
Work conditions (139)	
Increase	
Shift work (32)	*‘Shiftwork (sic) (in my current position), more friendly hours, would be great to create the work/life balance’*
Staffing (30)	*‘Better staffing would not go astray either’*
Personal support (29)	*‘support for emotional issues and tools to help support patients and their families though traumatic situations and the time to do that’*
	*‘As a first year nurse in my clinical area I have felt unsupported and thrown in the deep end. I understand that we are in busy times with little money but if there was anything i (sic) could change it would be how new graduate nurses are accepted into their placements. I feel many nurses need a big attitude adjustment about this and feel if we had a more positive response more young people like myself would stay in nursing’*
Nurse‐patient ratio (11)	*‘The nurse‐Patient Ratio. I see 1 nurse to 5‐6 pts as a safety risk. At this ratio I feel you are unable to provide safe appropriate (sic) care. This is very dissatisfying (sic) as a nurse’*
	*work load… to allow us to spend more one on one time with patients’*
Equipment (7)	*‘Management: get them to walk around the wards/unit, get them to help with a lift to realise the terrible/unsafe equipment we have to work with’*
Improve	
Work load (15)	*work load… to allow us to spend more one on one time with patients’*
Paper work (15)	*‘less time spent doing paper work and computer work and more time with patients’*
Salary increase (55)	*‘The PAY’*
	*‘Better pay and more benefits!!! Like health insurance etc.! Come on guys, get with the rest of the world!’*
	*‘Pay that reflects the hard work that we do and the impact we make in healthcare’*
Relationships with peers (34)	
Bullying (15)	*‘bullying attitude of other nurses, especially the older nurses who don't believe in the way we were trained’*
	*‘Nurses that bully other nurses, we need a great sense of team work to be able to do great things for our patients’*
Attitudes (19)	*‘Nursing attitudes towards junior nurses. It is a common misconception that junior nurses have no knowledge or skills’*
	*‘That being a new graduate nurse you are considered inherently incompetent even though your degree is all about proving you are. The difference between performing safely the fundamental competencies of nursing and not knowing about specific area policy and procedure is not recognised. Any lacking (sic) in obscure local policy is seen as reflection on your ability to be competent and safe at a fundamental level’*
Relationships with supervisors (22)	
Better management (18)	*That management recognized and respected the hard work that nurses do and showed appreciation!’*
	*‘Put people persons in management positions. It seems that nurse managers tend to be people who went into management to get away from patients and therefore don't have very good interpersonal skills’*
	*‘I really enjoy the job, but the politics and pettiness of some of the management gets bad at times and often makes you feel like it would be easier to just leave and find an easier profession’*
Decrease bullying by management (4)	*‘Better managers who spent the time to build up members of the team instead of cutting them down at the first opportunity (Horizontal violence)’*
	*‘The bullying of management that has made all of the experienced staff leave’*
Personal life (4)	*‘better work/life balance’*
	*‘The feeling(sic) of duty to the place, e.g. (sic) feeling guilty asking for time off when the children are sick’*
Category: motivation factors (26)	
Recognition (13)	*‘The respect from other health professional. The general lack of understanding (sic)of what the nursing profession is by the general public as well as other health’*
	*‘feel nurses are incredibly valuable (sic) within society & we are not always given the recognition we deserve’*
Work itself (7)	
Increase autonomy (4)	*‘more independence in practice’*
Decrease stress (3)	*‘just the stressful times’*
Advancement (5)	*‘more chance for promotion’*
Achievement (1)	*‘more availability postgraduate courses’*

Numbers in brackets = number of comments per category/subcategory.

### Maintenance factors

#### Work conditions

Most comments (139, 55% of all comments for maintenance factors) referred to the maintenance category of work conditions. The respondents would have liked to improve their working conditions by increasing or improving; Shift work (32 comments), staffing numbers (30 comments), personal support (29 comments), nurse‐patient ratio (11 comments) and equipment (7 comments) or decreasing/improving; work load (15 comments) and paper work (15 comments).

The most cited concerns regarded as resources necessary to improve work conditions were improved shift work patterns, increased nursing staff and increased personal support. In addition, respondents wished to see an increase in the nurse‐to‐patient ratio and improvements to the equipment that they work with. In addition to these, the need to decrease workloads and paper work were noted in equal measure as factors that needed to be changed and improved on.

#### Salary

Salary was commented on by 55 respondents, all of whom wanted to have nursing salaries increased for recognition of their educational level and skill and/or for retention purposes.

#### Relationships with peers

Thirty‐four comments were received which related with maintenance factor ‘relationships with peers’. Approximately half of the comments in this category were related with the need for other, mostly more experienced nurses, to show more appreciation for Generation Y nurses while the rest of the comments related with the need for bullying of nurses by their peers to stop.

#### Relationships with supervisors

Twenty two comments were associated with the category of ‘relationship with supervisors’. The majority of these comments (18) noted the need for the respondents to see an improvement in relationships with supervisors, namely those in management positions. However, the terms management, managers and corporate were all used to describe relationships with supervisors, so it was not clear from the comments which management personnel or positions in particular were being targeted, such as unit or ward charge nurses, service managers, hospital or corporate managers. Four comments were specifically related with the need for supervisors (unspecified) to decrease their bullying of nurses.

#### Personal life

The least number of comments (4) was received about the maintenance factor ‘personal life’. Changes that respondents would have liked to see were related with the need for a better work‐life balance. No comments were received about the Herzberg maintenance categories of status, security, relationships with subordinates, supervision, or company policy or administration.

### Motivation factors

#### Recognition

The most commented on motivation factor was the category of ‘recognition’, with 13 comments received. The respondents wished to see the nursing profession more recognized by the public and other members of the inter‐professional team for the work that nurses do. It was not clear from the comments that what the term ‘more recognition’ meant; however, five comments suggested that the public did not understand the role of the contemporary nurse.

#### Work itself

With regard to the motivation category ‘work itself’, seven comments were received, with a split between the need for nursing practice to offer more autonomy and the need for nursing to be less stressful.

#### Advancement

Five comments were received for the category ‘advancement’, with all respondents noting the need for more promotion opportunities.

#### Achievement

The one comment received for the motivation category ‘achievement’ noted the need for more postgraduate courses. No comments were received about Herzberg's motivation categories of responsibility or personal growth.

## Discussion

It is clear that the work of nursing is important to Generation Y nurses. As noted by Gardner *et al*., they view their work as ‘good work’ where by the needs of the patient are paramount. However, there is a tension between the delivery of this ‘good work’ and what factors these nurses would change in nursing work if they could. When asked to comment on ‘what one thing would you like to change about nursing’ responses were overwhelming skewed towards Herzberg's ‘push factors’. This is of concern to the profession given that the ongoing global nursing shortage means that nurse retention is already a top priority in most countries (Shaffer 2014). While these nurses enjoy their work, their commitment for a long term career is not evident (Jamieson *et al*. 2012). As such, this finding should also be a concern for the profession and issues surrounding nurses retention. Locally, the Nursing Council of New Zealand (2013) note that the nursing population is ageing at the same time as a future crippling supply shortage of nurses is predicted. This means that the retention of these Generation Y nurses is essential.

All respondents noted that there was room for improvement in most factors that are considered to be imperative if employees are to not feel dissatisfied with their work and therefore able to concentrate on obtaining and maintaining the satisfying motivators (Herzberg *et al*. 1959, Herzberg 2008). For example, the majority of respondents noted that work conditions needed addressing. The respondents expressed their concerns that work conditions such as poor shift work allocations impacted negatively on their health and contributed to increased levels of stress. West *et al*. (2007, 2009) noted that newly graduated nurses find the shift challenging, given their lack of tolerance towards a disrupted social life.

Further, new graduate nurses may be susceptible to shift work induced depression and burnout, key precursors or push factors from work (Navigate 2002, West *et al*. 2007). Such concerns may also negatively impact on the nurses’ sense of having a work‐ life balance. McCabe *et al*. (2003) also reported concern among nurses about the stressful nature of nursing work. Generation Y nurses surveyed by Clendon and Walker (2011) noted that they were not prepared as undergraduates for the emotional stress they encountered as registered nurses. Of concern, Clendon and Walker (2011) noted that Generation Y nurses who felt emotionally stressed were highly likely to leave nursing in the next twelve months. Others have also reported that nursing is becoming increasing stressful (World Health Organization 2006) with stress due to high workloads a key reason why nurses leave the profession (Tourangeau *et al*. 2009). Moreover, for nurses, decreased job satisfaction and an increase in adverse patient outcomes have been noted as a result of shift work induced stress and fatigue, as have increased personal injuries (Keller 2009, Barker & Nussbaum 2011).

Furthermore, the perceived lack of safe staffing levels and the lack of personal support have the potential, according to Herzberg (1968) and Lipkin and Perrymore (2009), to significantly contribute to dissatisfied employees. Moreover, some respondents noted that a high nurse‐patient ratio was of concern to them due to safety risks. These concerns are coupled with disquiet that high workloads and paper or computer work are barriers to being present at the bedside, which is where these nurses want to be (Jamieson 2012).

Given the extensive literature about members of Generation Y wanting mentorship (AH Revelations 2006, McCrindle 2006, Healy 2008, McCrindle & Pleffer 2008, Hershatter & Epstein 2010) and on‐going feedback, it is a concern for the profession that many of the respondents expressed their negative views about the lack of personal support in the workplace. As well, the reported incidences of poor relationships with peers due to bullying and negative attitudes of more experienced peers and poor relationships with supervisors are reflective of push factors that may entice these nurses to other work (Herzberg *et al*. 1959, Navigate 2002, Miller 2006, Moody & Pesut 2006, Black 2008).

Respondents were unanimous that their current salaries did not fairly reflect their work. Herzberg noted that a perceived unfairness of salary was highly likely to result in dissatisfied employees. Such dissatisfaction according to Herzberg contributes to push factors from work (Herzberg 1968).

In summary, while Generation Y nurses were happy with their career choice to enter the nursing profession, their views of nursing work and the changes that need to be made are of concern. The level of dissatisfaction of these nurses is well‐defined by their requests to change and improve work maintenance factors, such as working conditions. This should be of great concern for the profession given that Herzberg (1968, 2008) research has clearly demonstrated that workers concerns with hygiene‐maintenance factors are the very same factors that will push workers to other work. In other words, if Generation Y nurses perceive that nursing offers poor working conditions, and poor salary, coupled with tense relationships with peers and supervisors they are not likely to stay in the profession. Nursing cannot afford this situation to continue, given the global shortage of nurses and the increasing demands on nursing services.

### Study limitations

Accessing the Generation Y nurses via the Nursing Council of New Zealand limited the sample size because the majority of eligible participants had either not opted in to being considered for more surveys or they did not wish to be surveyed via email. Further, only surveying participants with an email address prevented those without email from participating. The self‐select nature of surveys may also add bias to the results. The sample size could be viewed as small, however, the American Association for Public Opinion Research (2011) have noted that ‘experimental comparisons have revealed few significant differences between estimates from surveys with low response rates and short field periods and surveys with high response rates and long field periods’ (para. 5).What is important is to ensure other measures of quality are included in the study.

This study was conducted over a 6 months from 2009–2010. Since that time, no new published research specific to Generation Y New Zealand nurses, other that already mentioned, has been located. However, the New Zealand Nurses Organisation has published two research reports since 2010 about New Zealand nurses in general (New Zealand Nurses Organisation 2011, Walker & Clendon 2013). Results from both reports confirm findings from this study. A 2011 survey of New Zealand nurses over the age of 30 years (*n* = 1076) noted that nurses agreed or strongly agreed that they were poorly paid and they disliked shift work. Further, the nurses felt strongly that their job satisfaction had been significantly reduced due to; high workloads, high patient acuity and high staff turnover (New Zealand Nurses Organisation 2011). A repeat of survey in 2013 of 1448 New Zealand Nurses, this time inclusive of nurse under the age of 30 years, noted an increase in concerns about poor moral. It was proposed by the researchers that this was due to even heavier workloads than those reported in 2011 (Walker & Clendon 2013).

## Conclusion

Given the ageing New Zealand nursing workforce couple with an ageing population and an increasing demand for health services it is clear that the retention of young Generation Y registered nurses in the healthcare workforce is essential. It is imperative that employers of nurses and government organisations responsible for nursing work force planning understand what push or pull factors are motivating these nurses to remain in, or exit from the profession with a view to developing strategies to address their concerns. If Generation Y New Zealand registered nurses are to remain in the workplace then the workplace needs to develop motivators to keep them there. If this does not occur the potential exists for the healthcare workplace to either be overwhelmed by dissatisfied workers which will be detrimental to both the nurses and patients or a workplace that is short of nurses.

## Conflict of interest

No conflict of interest has been declared by the authors.

## Author contributions

All authors have agreed the final version of this paper. Concept and design: IJ, RK, SW, CA. Acquisition of data: IJ. Analysis of data: IJ. Interpretation of data: IJ, RK, SW, CA. Drafting of the article: IJ. Review and critique: RK, SW, CA.

All authors have agreed on the final version and meet at least one of the following criteria [recommended by the ICMJE (http://www.icmje.org/recommendations/)]:

- substantial contributions to conception and design, acquisition of data, or analysis and interpretation of data;
- drafting the article or revising it critically for important intellectual content.
